# Evolutionarily Optimized Multi-Scale Gabor Modeling of Directional Lesion Texture in Dermoscopic Images for Interpretable Melanoma Classification

**DOI:** 10.3390/diagnostics16101430

**Published:** 2026-05-08

**Authors:** Raúl Santiago-Montero, Valentin Calzada-Ledesma, David Asael Gutiérrez-Hernández, Lucero de Montserrat Ortiz-Aguilar, Armando Mares-Castro, Luis Angel Xoca-Orozco, José de Jesús Flores-Sierra

**Affiliations:** 1 Tecnológico Nacional de México/IT de León, León 37290, Mexico; raul.santiago@leon.tecnm.mx (R.S.-M.);; 2 Tecnológico Nacional de México/ITS de Purísima del Rincón, Purísima del Rincón 36425, Mexico

**Keywords:** computer-aided diagnosis, skin cancer classification, melanoma, Gabor filter banks, differential evolution

## Abstract

**Background**: Melanoma is one of the most aggressive forms of skin cancer, making early and accurate diagnosis essential for improving patient outcomes. **Methods**: In this work, we propose an Evolutionary Gabor-based Melanoma Descriptor (Evo-GMD), a lightweight and interpretable approach designed under the principles of Frugal AI. The method integrates multi-scale Gabor filtering with Differential Evolution to automatically learn discriminative texture patterns using a reduced set of parameters. The proposed approach was evaluated on the PH2 dataset, achieving competitive performance (accuracy above 95%) while maintaining low computational complexity and full interpretability. To further assess its robustness, complementary experiments were conducted on the ISIC 2017 dataset, which presents higher variability, class imbalance, and heterogeneous lesion characteristics. **Results**: The results reveal that multiple methods—including handcrafted descriptors, convolutional neural networks, and transfer learning models—exhibit significant performance degradation or converge to trivial solutions under these conditions. This behavior highlights that increasing model complexity does not necessarily improve classification performance when data constraints are present. **Conclusions**: Overall, the findings demonstrate that the proposed method provides a robust and efficient alternative for melanoma classification in low-resource scenarios, where data availability, computational capacity, and interpretability are critical factors.

## 1. Introduction

Skin cancer remains one of the most prevalent and fastest-increasing malignancies worldwide, with risk factors including excessive exposure to ultraviolet radiation, genetic predisposition, and environmental factors. In Australia, one of the countries with the highest incidence rates, recent epidemiological reports indicate that more than 18,000 new cases are diagnosed annually, reflecting a sustained increase of approximately 25% over the last decade [1]. Similarly, in the United States, recent statistics report nearly 100,000 new cases per year, along with more than 7900 skin cancer-related deaths, underscoring the significant public health burden associated with this disease [2]. In Mexico, data from the National Institute of Statistics and Informatics (INEGI) identify skin cancer as one of the leading causes of hospital morbidity, ranking fifth overall, with a higher prevalence in men (7 out of 100 cases) compared to women (4 out of 100 cases) [3].

Among the various types of skin cancer, melanoma is particularly concerning. Although it ranks third in terms of the number of cases, it is responsible for 75% of skin cancer-related deaths [4,5]. The Skin Cancer Foundation emphasizes that patients with stage 1 melanoma (i.e., localized tumor) should receive treatment within 30 days of biopsy confirmation, as delays of 20 to 59 days could increase the risk by up to 5%, and a staggering 41% if delayed further. [4]. In clinical practice, this growing burden is compounded by the limited availability of dermatology specialists, particularly in public and resource-constrained healthcare systems, where timely access to expert evaluation is not always guaranteed. As a result, supportive Computer-Aided Diagnosis (CAD) tools have become increasingly relevant in assisting clinicians with the early identification of high-risk lesions and prioritizing cases requiring urgent intervention.

In clinical practice, the diagnosis of melanoma is commonly guided by visual assessment criteria such as the well-established ABCD rule, which evaluates skin lesions based on Asymmetry, Border irregularity, Color variation, and structural Diversity [6]. Owing to its clinical relevance, this rule has motivated extensive research aimed at translating dermatological assessment principles into automated image analysis frameworks for CAD. Most computational approaches inspired by the ABCD rule have focused on modeling global lesion characteristics, including shape irregularities, border properties, and overall color statistics. While these strategies have shown promise, they often rely on coarse quantifications of intensity distributions or global statistical measures, and tend to overlook the directional organization of lesion heterogeneity. This aspect, closely associated with asymmetric pigmentation and irregular tissue structures, is diagnostically relevant but has received comparatively limited attention in CAD systems. Moreover, modeling such directional patterns does not necessarily require full color information; grayscale intensity images suffice to capture the oriented textural variations that reflect underlying tissue disorganization.

Texture analysis plays a fundamental role in melanoma assessment, as malignant lesions typically exhibit irregular, heterogeneous, and directionally organized textural patterns, in contrast to benign lesions, which tend to present more uniform and isotropic texture distributions [7]. These directional patterns frequently arise from asymmetric tumor growth and heterogeneous tissue structures, and are routinely considered by dermatologists during visual inspection. Despite their clinical relevance, such directional texture attributes have received comparatively limited attention in automated melanoma analysis. Accurately capturing these patterns remains a central challenge in dermoscopic image analysis, as it requires descriptors capable of jointly modeling texture orientation and spatial heterogeneity while remaining robust to variations in illumination and lesion morphology.

To address these limitations, recent research in melanoma analysis has increasingly relied on DL approaches, which are capable of automatically learning complex feature representations from dermoscopic images. While DL-based models have achieved competitive performance in melanoma classification tasks [8,9], their effectiveness typically depends on the availability of large, well-annotated datasets, data augmentation techniques, and substantial computational resources. Moreover, the internal representations learned by these models are often difficult to interpret from a clinical perspective, which can limit their acceptance in diagnostic settings where transparency and explainability are essential.

In contrast, feature-based approaches remain highly relevant for modeling clinically meaningful lesion patterns, particularly in scenarios with limited data availability. By explicitly designing descriptors that reflect dermatological assessment criteria—such as texture orientation and spatial heterogeneity—these methods enable a direct interpretation of the features used for classification. Within this context, effective melanoma descriptors must be capable of capturing not only the presence of textural variability, but also its directional organization, as these oriented patterns are closely associated with asymmetric pigmentation and irregular tissue structures observed in malignant lesions.

Gabor Filter Banks (GFBs) provide a well-established feature-based framework that naturally fulfills these requirements by modeling localized patterns across multiple orientations and spatial frequencies [10]. In addition, employing GFBs at multiple spatial scales enables the capture of image features ranging from fine-grained textures to broader structural patterns, enhancing robustness to variations in lesion size and morphology. When applied to grayscale intensity images, multi-scale representations allow for the characterization of directional texture patterns in a manner that closely aligns with the visual assessment of lesion heterogeneity performed by dermatologists, while maintaining low computational complexity and interpretability. As such, Multi-Scale GFBs (MS-GFBs) constitute a principled foundation for the development of clinically interpretable melanoma descriptors.

However, the practical effectiveness of Gabor-based approaches is heavily influenced by their parameter configuration. The high dimensionality and continuous nature of this parameter space make manual design impractical, as it requires expert knowledge and extensive trial-and-error procedures that are not only time-consuming but also prone to suboptimal and irreproducible configurations. To address this problem, some recent approaches have proposed learning Gabor-like filters within DL frameworks such as Convolutional Neural Networks (CNN), often referred to as Gabor-learned or Gabor-inspired CNNs [11,12]. While these hybrid models allow for optimization of filter parameters based on data, they inherit the limitations of DL approaches. Consequently, many existing Gabor-based methods rely on fixed parameter settings or heuristics or integrate Gabor filters into deep architectures, which can limit their applicability and generalizability across different datasets and clinical conditions [8].

To address these challenges, this work presents a Gabor-based evolutionary framework for melanoma classification which adopts a Frugal AI perspective, that explicitly targets the multi-scale modeling of directional lesion texture within the Region of Interest (ROI) of skin lesions in dermoscopic images. Unlike previous approaches that employ Gabor filters primarily for generic texture analysis, the proposed framework is designed to capture clinically meaningful textural patterns associated with asymmetric pigmentation and heterogeneous lesion structure. Optimized MS-GFBs are employed to enhance discrimination while preserving interpretability and computational efficiency. Rather than relying on manual filter design or exhaustive grid-search strategies, the framework leverages Differential Evolution (DE) to adapt the frequency, orientation, and scale of Gabor filters according to the specific textural patterns of melanoma lesions. In this context, the evolutionary process is guided by the classification performance of a Support Vector Machine (SVM), enabling the discovery of filter configurations that emphasize diagnostically relevant textural patterns under limited data and computational constraints, and without data augmentation or external pretraining.

The proposed framework was evaluated on the dermatological dataset PH2 and compared against traditional handcrafted descriptors and representative DL approaches. Experimental results indicate that the proposed method improves classification performance compared to the tested approaches, while reducing computational cost and limiting dependence on large annotated datasets. These findings suggest that the proposed framework constitutes a practical and explainable alternative for AI-assisted melanoma analysis, particularly in scenarios with constrained data availability and computational resources.

The manuscript is organized as follows. Section 2 reviews previous studies on descriptors and melanoma classification. Section 3 introduces the proposed evolutionary framework for modeling directional lesion texture. Section 4 describes the experimental design, including dataset preparation and parameter configuration. Section 5 and Section 6 present and discuss the experimental results. Finally, Section 7 concludes the paper and outlines directions for future research.

## 2. Related Work

The development of CAD tools for melanoma classification has evolved into two main approaches: data-driven DL models and interpretable feature-based methods. While DL dominates recent literature due to its baseline performance, feature-based approaches remain indispensable for clinical confidence and resource-limited settings.

CNNs have achieved state-of-the-art accuracy on large datasets such as HAM10000, with performance exceeding 94% in controlled settings [9]. Despite their success, these models face significant barriers to clinical adoption. For example, reliance on massive datasets limits applicability in medical diagnostics, where labeled data is scarce and laborious to curate [8]. Moreover, DL architectures demand substantial computational resources for training and inference, a requirement incompatible with low-resource settings or real-time diagnostics [13]. One way to address the lack of computational resources is through transfer learning; for example, in [14], a VGG-16 architecture is improved, achieving competitive results for skin cancer classification. However, their black-box behavior undermines physician trust, rendering decision-making processes potentially opaque [15], not to mention that their robustness under varying imaging conditions (e.g., illumination variations, device heterogeneity) also remains a challenge, particularly in decentralized healthcare systems [5].

Feature-based approaches, in contrast, prioritize interpretability and computational efficiency, closely aligning with dermatologists’ diagnostic workflows. The ABCD rule (Asymmetry, Border irregularity, Color variation, and Different structures) has been instrumental in this regard, inspiring systems that quantify malignancy risk through manually designed features. For example, the Total Dermoscopic Score translates the ABCD criteria into a weighted formula to stratify lesions [6]. These methods are notable for their transparency, as clinicians can attribute decisions to specific features such as color heterogeneity or border irregularity. However, their performance depends on the subjective design of the features. For example, the quantification of color variation (a critical indicator of malignancy) often relies on histogram statistics in RGB/HSV spaces [16] or clustering-based diversity metrics [17], which require laborious tuning and expert validation.

From a texture analysis perspective, techniques such as Haralick and Local Binary Patterns (LBP) are used to extract spatial patterns in skin diseases [9,18]. However, these struggle to capture lesion heterogeneity across multiple scales. Similarly, color quantification methods range from intensity histograms [19] to hybrid descriptors such as Color Local Directional Patterns [20], which combine edge and texture features. While these approaches are computationally low compared to DL, they often rely on fixed thresholds or hand-crafted rules, limiting adaptability to lesion-specific variations. For example, Seeja et al. [17] proposed an 8×M×N neighborhood matrix to encode color–texture relationships, but the resulting 18 features required manual validation to ensure discriminative power, a process prone to bias and scalability issues.

Developing algorithms by hand often presents three fundamental limitations: First, feature design relies heavily on domain expertise and trial and error, which carries the risk of obtaining suboptimal representations. Bakheet et al. [21], for example, generated a 2.6 million-element Gabor feature vector, highlighting the inefficiency of unoptimized descriptors. Second, parameter sensitivity affects techniques such as Gabor filters, where tuning frequency, orientation, and bandwidth become a high-dimensional optimization problem typically addressed by grid-search or expert intuition [22]. Third, rigid descriptors such as Haralick or histograms do not accommodate lesion-specific variations in scale or morphology, reducing generalizability across diverse populations.

Recent advances in skin cancer analysis have explored hybrid methodologies that combine Gabor filters with machine learning models to improve diagnostic accuracy. For example, Al-Nuaimy et al. proposed a system that leverages Gabor features extracted from terahertz images, achieving 94% accuracy using an Artificial Neural Network [23]. While their approach demonstrates the utility of Gabor filters in capturing spatial frequency information, it relies on fixed filter parameters, limiting adaptability to lesion-specific textures. Similarly, Benyahia et al. presented a Gabor Convolutional Network that integrates Gabor filtering with CNN to reduce feature extraction burden and improve classification performance, achieving 96% accuracy on dermoscopic images [24]. Similar to this proposal in [24], a hybrid approach is provided to fuse interpretable Gabor responses into CNNs. Although these hybrid models outperform traditional CNNs, they inherit the computational complexity and reliance on massive data to perform DL, making them less suitable for resource-constrained settings. In a different approach, Alom et al. combined the Gabor wavelet transform with an SVM optimized via Particle Swarm Optimization, achieving an accuracy of 87% [25]. While their method improves interpretability and reduces feature dimensionality, it still requires manual tuning of Gabor parameters and extensive preprocessing. Gabor filters, which combine spatial and frequency domain analysis, are widely used in dermatological CAD tools [22]. Still, their effectiveness depends on parameter tuning, a process rarely automated in the work as mentioned above.

These studies underscore the potential of Gabor filters in skin lesion analysis but highlight critical gaps in adaptive parameter optimization and computational efficiency, which our work addresses through evolutionary algorithms. While deep learning models have shown strong performance in large-scale settings, their effectiveness often depends on data augmentation and pretraining. In imbalanced or limited datasets, these requirements may restrict their applicability, motivating the exploration of lightweight and interpretable alternatives.

## 3. Methodology and Materials

This section describes the methodological and material components of the proposed evolutionary framework for melanoma characterization. First, the representation of skin lesions using MS-GFBs is introduced, detailing how textural information is extracted from lesion ROIs. Next, the Evolutionary Gabor-based Melanoma Descriptor is presented, including the formal definition of the associated optimization problem and the evolutionary process employed to optimize the Gabor filter parameters. Together, these subsections provide a comprehensive description of the feature extraction and optimization procedures underlying the proposed approach.

### 3.1. Description of Skin Lesions Using MS-GFBs

From a dermatoscopic image analysis perspective, beyond purely morphological characteristics, the textural variability within the lesion constitutes a key diagnostic factor, particularly when its spatial and directional organization is taken into account. To effectively characterize these patterns, MS-GFBs provide a powerful tool for extracting multiscale and multiorientation representations closely related to lesion heterogeneity.

To ensure that the MS-GFBs focus on clinically relevant information, each dermatoscopic image analyzed is converted to grayscale and cropped to its ROI to emphasize intensity variations and ensure a consistent input representation for Gabor filtering. The use of grayscale images allows the model to focus on structural and directional texture patterns, which are effectively captured through multi-scale Gabor filtering. This design choice also reduces computational complexity and aligns with the Frugal AI paradigm.

Let I(x,y) denote the image resulting from the grayscale ROI cropping process, where each pixel (x,y) represents an intensity value. The image can be represented as an array of intensity values, defined as follows:

(1) I=I(x,y)∣x=1,…,M;y=1,…,N

where *M* and *N* denote the spatial dimensions of the image.

To analyze the textural variability within I(x,y), an MS-GFB composed of a set of $N_{f}$ Gabor filters ${\left\{g_{j}\left(x,y\right)\right\}}_{j=1}^{N_{f}}$ is used, where each filter is defined by a specific set of parameters.

Each Gabor filter is defined as:

(2) $$g_{j}\left(x,y\right)=\exp\left(-\frac{x^{\prime 2}+\gamma_{j}^{2}y^{\prime 2}}{2\sigma_{j}^{2}}\right)\sin\left(2\pi \frac{x^{\prime }}{\lambda_{j}}+\psi_{j}\right)$$

where

- $x^{\prime }=x\cos\theta_{j}+y\sin\theta_{j}$ and $y^{\prime }=-x\sin\theta_{j}+y\cos\theta_{j}$, defining a rotation of the filter according to its orientation $\theta_{j}$;
- $\lambda_{j}$ is the wavelength of the sinusoidal component, controlling the scale of the pattern the filter responds to;
- $\sigma_{j}$ and $\gamma_{j}$ determine the spatial extent of the Gaussian envelope, with $\sigma_{j}$ controlling its size and $\gamma_{j}$ adjusting its aspect ratio;
- $\psi_{j}$ is the phase offset of the sinusoid;
- $m_{j}$ is the size of the square window used for convolution, defining the local neighborhood considered for feature extraction.

These parameterized filters, capturing orientation ($\theta_{j}$), wavelength ($\lambda_{j}$), spatial extent ($\sigma_{j},\gamma_{j}$), and window size ($m_{j}$), are then applied to the image *I* through a two-dimensional convolution, denoted by ∗. This windowed convolution produces the response $R_{j}\left(x,y\right)$ for each filter *j*, capturing localized texture variations. The choice of $m_{j}$ provides a trade-off between spatial resolution and the ability to capture broader structural patterns, ensuring that the resulting responses are both discriminative and suitable for subsequent evolutionary optimization.

Likewise, the convolution operation is formally defined as:

(3) $$R_{j}\left(x,y\right)=I\left(x,y\right)*g_{j}\left(x,y;m_{j}\right),\;j=1,\ldots ,N_{f}$$

Here, $m_{j}$ denotes the window size of the *j*-th Gabor filter, corresponding to its spatial scale. By employing filters at multiple scales, this approach enables the extraction of image features at varying levels of spatial resolution, enhancing the representation of both fine and coarse structures.

Once the filter responses $R_{j}\left(x,y\right)$ are obtained, first-order statistical features—namely the mean ($\mu_{j}$) and standard deviation ($\sigma_{j}$)—are extracted from each convolutional response to characterize the skin lesion. The mean captures the average energy of the texture patterns emphasized by a given Gabor filter, while the standard deviation quantifies the dispersion of these responses, reflecting the degree of structural heterogeneity within the images. Together, these statistics provide a compact yet informative representation of lesion appearance, enabling effective discrimination while preserving interpretability and computational efficiency.

Formally, the mean value of the response $R_{j}\left(x,y\right)$ corresponding to the *j*-th Gabor filter is computed as:

(4) $$\mu_{j}=\frac{1}{MN}\sum_{x=1}^{M}\sum_{y=1}^{N}R_{j}\left(x,y\right)$$

where $\mu_{j}$ represents the average intensity (or energy) of the filtered response, and *M* and *N* denote the spatial dimensions of the image.

Similarly, the standard deviation of the response is calculated as:

(5) $$\sigma_{j}=\sqrt{\frac{1}{MN}\sum_{x=1}^{M}\sum_{y=1}^{N}{\left(R_{j}\left(x,y\right)-\mu_{j}\right)}^{2}}$$

where $\sigma_{j}$ quantifies the variability of the texture pattern detected by the *j*-th filter. The square root ensures that the resulting value is expressed in the same units as the image intensity.

For each filter *j*, the values $\mu_{j}$ and $\sigma_{j}$ can be included in a feature vector F used for skin lesion classification:

(6) $$F=[\mu_{1},\sigma_{1},\mu_{2},\sigma_{2},\ldots ,\mu_{N_{f}},\sigma_{N_{f}}]$$

The resulting feature vector F encodes information about the directional lesion texture by summarizing the responses of the optimized MS-GFBs. These features quantify the presence and heterogeneity of structural patterns within the ROI, which are strongly associated with clinically relevant differences between benign and malignant lesions.

### 3.2. Evolutionary Gabor-Based Melanoma Descriptor

The proposed method is designed under the principles of Frugal AI, prioritizing low computational complexity, interpretability, independence from data augmentation, and training from scratch without relying on external datasets. These characteristics differentiate the proposed approach from deep learning (DL) methods, which typically depend on large-scale pretraining and extensive data augmentation strategies.

In line with these design principles, the optimization process is carried out using DE, a population-based metaheuristic widely adopted for continuous optimization problems [26]. Unlike other classical evolutionary algorithms, DE employs mutation and crossover operators to effectively diversify the population and promote exploration of the search space, reducing the risk of premature convergence. Additionally, DE requires only a small number of hyperparameters, making it a simple and computationally efficient technique to implement.

In this work, we propose to evolve MS-GFBs to generate a highly discriminative descriptor for melanoma images. The evolved descriptor, which adapts the parameters of Gabor filters for optimal feature extraction, is referred to as Evolutionary Gabor-based Melanoma Descriptor (Evo-GMD).

In general, an initial population of Evo-GMDs is generated, and for each descriptor, features are extracted from a dataset of labeled skin lesion images (*I*), cropped according to their ROI. The resulting feature vectors are organized into a dataset, which is subsequently classified using an SVM with a cross-validation scheme. The classification performance, measured in terms of average accuracy, is used to calculate the fitness value for each Evo-GMD, defined in this work as the classification error. Based on this fitness evaluation, the evolutionary operators of DE (mutation, crossover, and selection) are applied to generate a new population of Evo-GMDs. This process is iteratively repeated until the stopping criteria are met. Finally, the framework outputs the best-performing Evo-GMD obtained during the evolutionary process.

**Figure 1:**
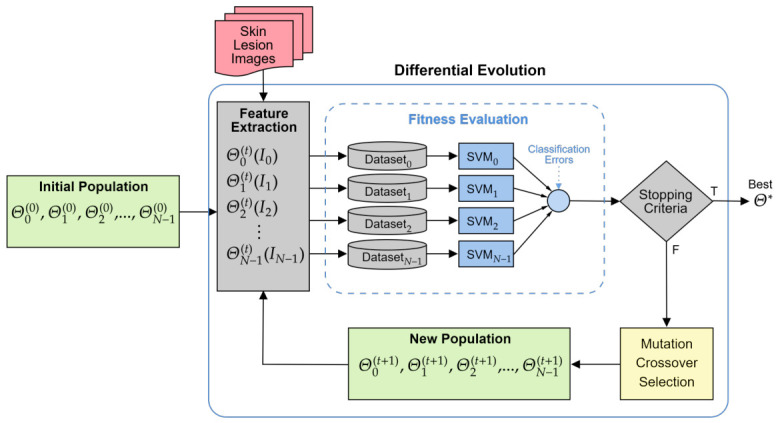
Evolutionary Gabor-based Melanoma Descriptor Methodology.

An overview of the complete proposed methodology is illustrated in Figure 1.

#### 3.2.1. Definition of the Optimization Problem

In DE, an Evo-GMD encodes the complete parameterization of a MS-GFB defined by a set of $N_{f}$ Gabor filters, where each depends on six parameters $\left\{m_{j},\sigma_{j},\theta_{j},\lambda_{j},\gamma_{j},\psi_{j}\right\}$, which control the shape and response of the filter. The dimensionality of the optimization problem increases proportionally with the number of filters included in the bank. Furthermore, each filter parameter is defined over a distinct domain, specified by its corresponding lower and upper bounds, which increases the complexity of the optimization problem. Under this formulation, an Evo-GMD at generation *t* is formally represented as:

(7) $$\Theta_{i}^{\left(t\right)}=\overset{N_{f}}{\underset{j=1}{⋃}}\left\{m_{j},\sigma_{j},\theta_{j},\lambda_{j},\gamma_{j},\psi_{j}\right\}$$

Thus, the objective function is defined to minimize the classification error E(·) associated with $\Theta_{i}^{\left(t\right)}$. For this purpose, a cross-validation scheme is employed on the dataset, ensuring that class proportions are preserved across *K* folds. In each *k* fold, the SVM is trained using the features extracted with $\Theta_{i}^{\left(t\right)}$ and evaluated on the corresponding test subset. The fitness value is then computed as the complement of the average classification accuracy across all folds, with respect to the ideal value of perfect accuracy:

(8) $$E\left(\Theta_{i}^{\left(t\right)}\right)=1-\frac{1}{K}\sum_{k=1}^{K}{\mathrm{Acc}}_{k}$$

where ${\mathrm{Acc}}_{k}$ denotes the classification accuracy obtained in the *k*-th fold of the cross-validation procedure. Consequently, the evolutionary optimization problem can be formally expressed as the minimization of the average classification error across all folds, leading to the following objective:

(9) $$\Theta^{*}=\arg\min_{\Theta }\left(1-\frac{1}{K}\sum_{k=1}^{K}{\mathrm{Acc}}_{k}\right)$$

This optimization strategy promotes the selection of Evo-GMDs that consistently yield high classification performance across multiple training-validation partitions, thereby reducing sensitivity to data partitioning and mitigating overfitting.

#### 3.2.2. Evolutionary Process

At the beginning of the evolutionary process, a population of *N* random $\Theta_{i}^{\left(t\right)}$ is generated within the specified domains.

For each $\Theta_{i}^{\left(t\right)}$, a mutated vector $V_{i}^{\left(t\right)}$ is generated using the scheme DE/best/1/bin as shown in the following equation:

(10) $$V_{i}^{\left(t\right)}=\Theta_{best}^{\left(t\right)}+F\cdot \left(\Theta_{a}^{\left(t\right)}-\Theta_{b}^{\left(t\right)}\right)$$

where *F* is a scaling factor controlling the magnitude of the mutation, $\Theta_{best}^{\left(t\right)}$ is the solution with the best fitness, and $\Theta_{a}^{\left(t\right)}$ and $\Theta_{b}^{\left(t\right)}$ are randomly chosen solutions:

Then, a candidate $U_{i}^{\left(t\right)}$ is generated by recombining $\Theta_{i}^{\left(t\right)}$ and $V_{i}^{\left(t\right)}$ with crossover probability CR:

(11) $$U_{i,j}^{\left(t\right)}=\left\{\begin{array}{cc} V_{i,j}^{\left(t\right)}, & \mathrm{if}\;rand_{j}\left(0,1\right)\leq CR\;\mathrm{or}\;j=j_{\mathrm{rand}}\\ \Theta_{i,j}^{\left(t\right)}, & \mathrm{otherwise} \end{array}\right.$$

Since recombination may generate values outside the predefined parameter limits, rescaling is applied to ensure each parameter remains within its valid domain. The rescaling for each parameter of a candidate solution is performed as follows:

Let $x_{j}$ be an element of $\Theta_{i}^{\left(t\right)}$, with lower bounds $L_{j}$ and upper bounds $U_{j}$. The rescaling is performed as follows:

(12) $$x_{j}=\left\{\begin{array}{cc} U_{j}-\left|x_{j}\;\mathrm{mod}\;\left(U_{j}-L_{j}\right)\right|, & \mathrm{if}\;x_{j}<L_{j}\\ L_{j}+\left|x_{j}\;\mathrm{mod}\;\left(U_{j}-L_{j}\right)\right|, & \mathrm{if}\;x_{j}>U_{j}\\ x_{j}, & \mathrm{other}\;\mathrm{case} \end{array}\right.$$

where the modulus operator is used to reflect values that are outside the bounds within the allowed range. This strategy ensures that any solution generated by the evolutionary algorithm remains within the valid search space without the need to directly truncate values, which better preserves the diversity of the population.

Finally, the objective function is evaluated, and the best solution is selected according to:

(13) $$\Theta_{i}^{\left(t+1\right)}=\left\{\begin{array}{cc} U_{i}^{\left(t\right)}, & \mathrm{if}\;E\left(U_{i}^{\left(t\right)}\right)<E\left(\Theta_{i}^{\left(t\right)}\right)\\ \Theta_{i}^{\left(t\right)}, & \mathrm{otherwise} \end{array}\right.$$

This process is repeated until the best Evo-GMD ($\Theta^{*}$) is obtained, which satisfies one of the following stopping criteria: (1) that the fitness function reaches a set error, or (2) that a predefined number of iterations is met.

## 4. Experimental Design

This section details the proposed experimental design, which objectively and reproducibly evaluates the performance of the Evo-GMD for classifying skin lesions. To this end, a two-stage experimental design was defined that allows, firstly, the optimization of the descriptor parameters without introducing biases arising from the use of test data, and secondly, the evaluation of its generalizability to independent data. Furthermore, the design includes a fair comparison with widely used reference methods in the literature, employing consistent data partitioning and classification configurations for all evaluated approaches.

### 4.1. Dataset Preparation

The PH2 dataset [27] is a collection designed for the research and evaluation of automatic diagnostic techniques in dermatology, particularly for classifying skin lesions from dermatoscopic images. This dataset is commonly used in studies focused on melanoma classification, a type of skin cancer, as well as other dermatological conditions.

The dataset comprises 200 dermatoscopic images, each approximately 765×575 pixels, corresponding to different skin lesions. Each image includes detailed annotations made by expert dermatologists and its binarized ROI. They are classified into three main categories with varying numbers of cases: 80 common nevi, 80 atypical nevi, and 40 melanomas.

This article focuses on two types of skin lesions: melanomas and common nevi. The decision to focus on these is based on the difficulty of distinguishing between malignant (melanoma) and benign (common nevus) skin lesions, a crucial process for early patient prognosis. Atypical nevi tend to grow more slowly and are less prone to metastasis, making their diagnosis less urgent compared to melanoma. By focusing on melanoma and common nevi, this study addresses the most critical and complex aspect of skin lesion classification, where diagnostic errors can have serious consequences.

To ensure consistent feature extraction, all images and ROIs in the dataset were rescaled to 720×480 pixels, adopting a standard 4:3 aspect ratio. Likewise, the dataset was split into two parts: train (80%) and test (20%). This strategy preserves the level of detail necessary for texture analysis, allowing the algorithms to operate under homogeneous spatial conditions without significant loss of structural information, while also balancing visual quality with computational efficiency.

**Figure 2:**
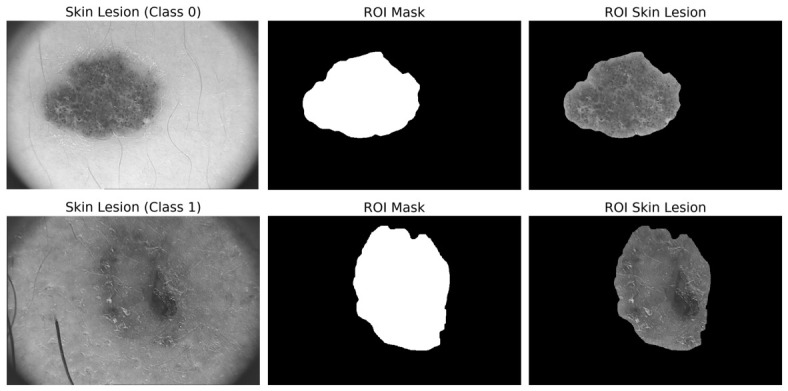
Representative images from the PH2 dataset.

Figure 2 illustrates two representative examples of skin lesion images: common nevus (labeled as Class 0) and melanoma (labeled as Class 1), their corresponding ROI, and the resulting image obtained after ROI cropping.

### 4.2. Experimental Protocol

The experimental protocol was designed to rigorously evaluate the performance of the proposed approach, ensuring a clear separation between the optimization process and the final evaluation on independent data. To this end, the experimental design was divided into two complementary stages. In the first stage, the parameters of the Evo-GMD were optimized using only the train set, while in the second stage, the generalizability of the optimized descriptor was evaluated on the testing set, as well as its comparative performance against different reference methods. This design avoids biases arising from the misuse of test data during optimization and guarantees a fair comparison between all the evaluated algorithms.

#### 4.2.1. Stage 1: Optimization of the Evo-GMD

For these experiments, the quality of each Evo-GMD is measured using the classification error obtained by a stratified SVM under a five-fold cross-validation scheme. This scheme allows for a robust estimation of the Evo-GMD performance, maintaining a balanced distribution of classes in each fold and reducing the risk of overfitting during the optimization process.

To analyze the stability and consistency of the evolutionary process, 31 independent runs of the DE algorithm were performed, each starting with a different random population. From the results obtained, descriptive statistics of the performance were calculated, including the mean, median, standard deviation, as well as the minimum and maximum values of the classification error. Finally, three representative Evo-GMDs were selected: the highest performing (best), the lowest performing (worst), and the median performing. These Evo-GMDs were stored for later analysis and evaluation in the next stage.

#### 4.2.2. Stage 2: Evaluation on the Testing Set and Comparison with Reference Methods

In this stage, the best, worst, and median performing Evo-GMDs obtained in Stage 1 are used independently to describe and classify the test set, which was not used at any point in the optimization process. For each case, and given that the dataset is unbalanced, a stratified SVM was trained using the entire training set, without applying cross-validation. Subsequently, the model was evaluated on the test set to estimate the actual generalization of the Evo-GMD.

Additionally, to contextualize the performance of the proposed approach, an experimental comparison was carried out with various reference methods widely used in the literature. These include traditional texture-based descriptors, as well as DL-based approaches, specifically: Haralick + SVM, LBP Uniform + SVM, a simple baseline CNN, a texture-based CNN, and the pre-trained VGG-16 architecture.

All algorithms were evaluated using the same train and test sets, and, in the case of the traditional descriptors, the same SVM configuration was used to ensure a fair comparison. Furthermore, the performance of all methods was quantified using standard metrics for classification problems, namely: Accuracy, Precision, Recall, and F1-score. This set of metrics allows us to evaluate not only the overall accuracy of the model, but also its ability to correctly identify positive cases, which is particularly relevant in the context of melanoma classification.

It is important to mention that the comparison methods were selected to cover a representative spectrum of approaches used in the literature for skin lesion classification. The Haralick and LBP Uniform descriptors were included as classic texture-based references, widely used for their interpretability and low computational cost. CNNs were considered to evaluate the performance of DL-based approaches at different levels of complexity, including a baseline CNN trained from scratch, a more sophisticated texture-based CNN architecture, and the pre-trained VGG-16 network, which is a well-established benchmark in medical image analysis. This selection allows for a balanced comparison between traditional and data-intensive methods under a common experimental framework.

### 4.3. Parameter Configuration

To ensure the reproducibility of the experiments and a fair comparison between the different approaches evaluated, all algorithms were implemented using carefully selected parameter configurations that favor generalization and avoid overfitting. In all cases, the same train and test sets were used, and when an SVM was used as the classifier, an identical configuration was maintained for all descriptors.

#### 4.3.1. DE and MS-GFB Hyperparameters

In DE, each Evo-GMD in the population encodes an MS-GFB of $N_{f}=4$ Gabor filters, where each filter is described by six parameters, resulting in a 24-dimensional decision vector. DE was configured with a population size of 30, a mutation factor F=0.8, a crossover rate CR=0.9, and a maximum of 30 iterations, using a convergence threshold of $1\times 10^{-6}$ as the stopping criterion. The values of *F* and CR were selected to provide a good balance between exploration and exploitation, promoting population diversity while enabling efficient convergence in continuous, high-dimensional optimization problems. The number of iterations was fixed at 30 based on preliminary experiments, which showed that increasing the number of generations did not produce significant improvements in the objective function while substantially increasing the computational cost. Finally, the domain for the Gabor filter parameters was defined as $m_{j}\in \left[1,20\right]$, $\sigma_{j}\in \left[1,5\right]$, $\theta_{j}\in \left[0,\pi \right]$, $\lambda_{j}\in \left[1,10\right]$, $\gamma_{j}\in \left[0,1\right]$, and $\psi_{j}\in \left[0,2\pi \right]$, covering a broad spectrum of spatial frequencies, orientations, and aspect ratios.

#### 4.3.2. SVM Hyperparameters

For this experimental protocol, an SVM with a linear kernel and a regularization parameter C=1 was used. This configuration was kept constant for all descriptors to ensure a fair comparison.

The choice of a linear kernel is based on two main considerations: (1) the descriptors used generate high-dimensional representations, where a linear hyperplane is usually sufficient to separate the classes, and (2) a linear model improves interpretability and reduces the risk of overfitting. The value of C=1 represents a standard compromise between classification margin and error, widely used in the literature when specific hyperparameter optimization is not performed.

#### 4.3.3. Classical Texture Descriptors Parameters

For the reference methods based on classical descriptors, widely reported standard configurations were used as shown in Table 1.

These configurations allow the capture of local patterns (LBP) and second-order statistical relationships (Haralick), serving as interpretable and widely accepted baselines in biomedical applications.

#### 4.3.4. Convolutional Neural Networks Parameters

To compare the proposed approach with DL methods, three architectures of increasing complexity were evaluated: the baseline CNN, the texture-based CNN, and the pre-trained VGG-16 model.

The baseline CNN was trained from scratch, representative of compact architectures used with small datasets. The texture-based CNN incorporates regularization and normalization mechanisms to improve generalization in texture analysis. Finally, VGG-16 was included as a reference for transfer learning, widely used in dermatological studies, allowing the proposed approach to be compared against high-capacity pre-trained models. Table 2 shows the complete parameter settings for all implemented DL approaches.

### 4.4. Experiments on ISIC 2017 Dataset

The International Skin Imaging Collaboration (ISIC) archive is one of the largest publicly available repositories of dermoscopic images, widely used to evaluate automated skin lesion analysis methods [28]. It has been the basis of multiple benchmark challenges, each focusing on tasks such as lesion segmentation, disease classification, and attribute detection.

In this work, the ISIC 2017 dataset was selected for complementary experimentation. This choice is motivated by two main factors. First, ISIC 2017 provides both classification labels and corresponding lesion segmentation masks, allowing the extraction of well-defined Regions of Interest (ROIs), which are essential for the proposed texture-based approach. In contrast, subsequent ISIC datasets do not consistently provide validated segmentation masks, making them less suitable for methods that rely on precise lesion localization. Second, ISIC 2017 offers a suitable balance between dataset size and annotation quality, enabling the evaluation of generalization under more challenging conditions than PH2.

The dataset contains a total of 2000 training images and 600 test images, originally labeled into three diagnostic categories: melanoma, nevus, and seborrheic keratosis. For this study, the problem was reformulated as a binary classification task by grouping nevi and seborrheic keratosis into a single non-melanoma class. This results in a significantly imbalanced distribution, with approximately 19% melanoma and 81% non-melanoma samples in both training and test sets.

To ensure consistency with the experimental setup used for the PH2 dataset, the ISIC 2017 images were preprocessed following the same pipeline. Specifically, lesion ROIs were extracted using the provided segmentation masks, converted to grayscale, and resized to a fixed resolution of 720×480 pixels. This standardized preprocessing allows a fair comparison between datasets while preserving the structural and textural characteristics required by the proposed method.

Despite this controlled preprocessing, ISIC 2017 introduces several challenges not present in PH2. The inclusion of seborrheic keratosis increases the heterogeneity of the non-melanoma class, making the classification task more complex. Additionally, the dataset exhibits significant variability in acquisition conditions, including differences in illumination, presence of artifacts such as hair and shadows, variations in skin tone, and inconsistencies in lesion scale and contrast. These factors contribute to a more realistic and challenging classification scenario.

The dataset is originally divided into training, validation, and test subsets. However, for the purposes of this study, only the training and test sets were used. This decision is aligned with the proposed evolutionary framework, which does not rely on a separate validation set for hyperparameter tuning. Introducing the validation set into the training process would create inconsistencies when comparing against models that explicitly depend on validation-based optimization.

Finally, it is important to note that all classification algorithms evaluated on ISIC 2017 were applied using exactly the same configuration as in the PH2 experiments. No additional tuning, data augmentation, or architecture modification was performed. This decision ensures methodological consistency and allows a direct assessment of how each approach generalizes under more complex and imbalanced conditions.

## 5. Results

### 5.1. Stage 1 Results

**Figure 3:**
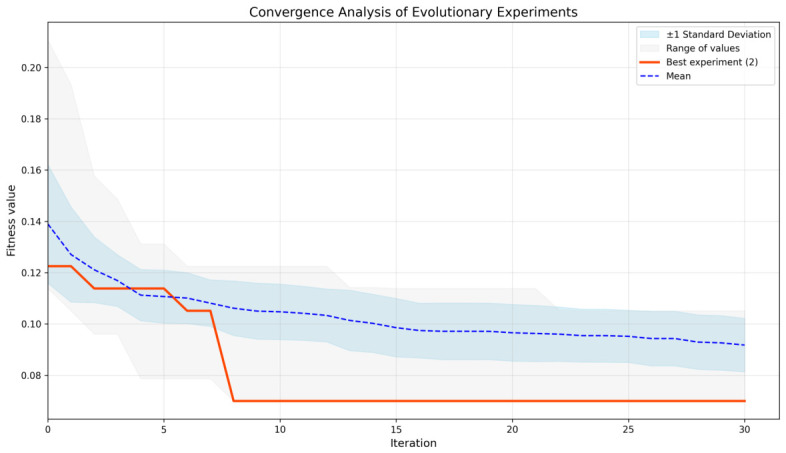
Convergence Graph of 31 Independent Experiments Using DE.

This section reports the results obtained in the first stage of the experimental protocol, which focuses on the Evo-GMD optimization. Figure 3 illustrates the convergence behavior of the fitness function across 31 independent runs, providing insight into the stability and consistency of the evolutionary process. Additionally, Table 3 summarizes the descriptive statistics of the fitness values, allowing a quantitative assessment of the variability and central tendency of the solutions obtained.

After conducting 31 independent runs, consistent and promising results were obtained with the proposed fitness function.

Figure 3 highlights the best-performing experiment (Experiment 2), indicated by the red curve. During the initial iterations, the fitness function exhibits a gradual decrease; however, after approximately five iterations, a more pronounced minimization trend can be observed, reaching fitness values below 0.08. Regarding the dispersion of results, both the range of fitness values and the standard deviation are higher at the early stages of the evolutionary process and progressively decrease after iteration 5, indicating increased stability. Furthermore, the mean fitness value consistently shows a decreasing trend across all experiments, reflecting a systematic convergence behavior.

Descriptive statistics of the fitness values are summarized in Table 3. The mean fitness was 0.0918, while the median, which provides a robust measure of central tendency, was 0.0960. These results indicate that most solutions are concentrated within a relatively narrow range, suggesting stable convergence of the evolutionary process. The standard deviation of 0.0106 further confirms moderate variability across experiments, implying that the proposed optimization scheme yields consistent and replicable solutions under the defined experimental conditions.

**Figure 4:**
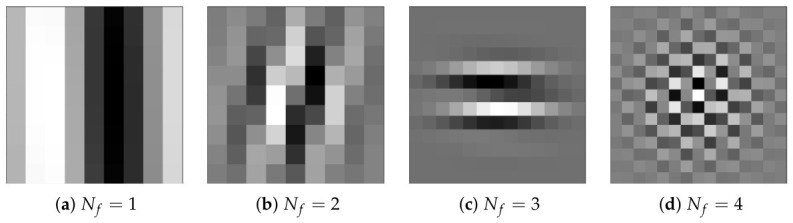
MS-GFB corresponding to the Evo-GMD with median performance.

**Figure 5:**
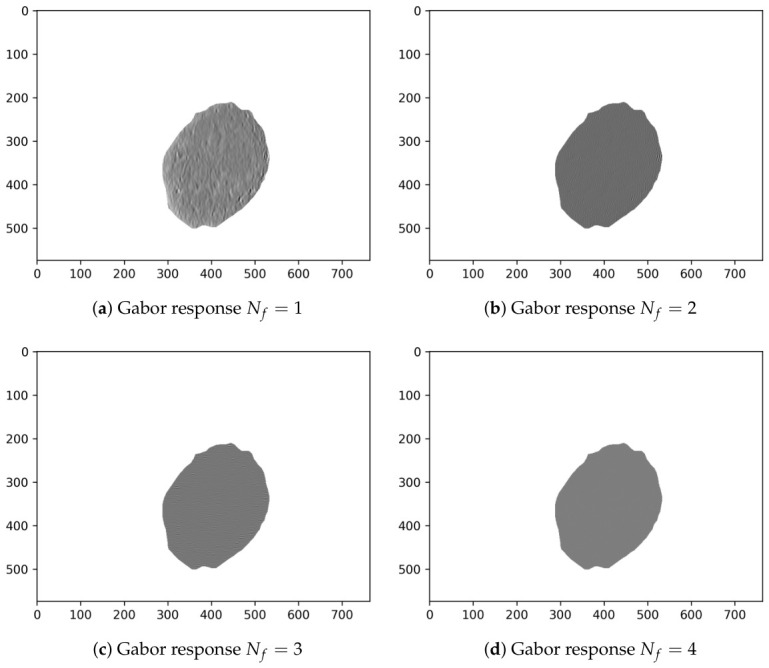
Gabor responses of the Evo-GMD with median performance applied to a common nevus image.

Finally, Table 4 shows the Evo-GMD parameters found by DE corresponding to the median, best, and worst fitness values. Likewise, Figure 4 visually shows an example of the Gabor filter corresponding to the Evo-GMD with medium performance, and Figure 5 shows the Gabor responses for each filter, revealing exactly what happens during the image filtering process. This feature makes Evo-GMD interpretable.

### 5.2. Stage 2 Results

This section reports the results obtained in the second stage of the experimental protocol, where the median, best, and worst Evo-GMDs were compared against classical texture descriptors and several DL approaches, using identical train and test partitions to ensure a fair and unbiased comparison.

Table 5 summarizes the classification performance in terms of Accuracy, Precision, Recall, and F1-score. Among the traditional descriptors, Haralick and LBP achieved moderate accuracy values (0.7619); however, both methods exhibited notably low recall, particularly in the case of Haralick (0.2857). This behavior indicates a strong bias toward the majority class, which is reflected in their reduced F1-scores and suggests limited robustness for lesion classification under the evaluated conditions.

With respect to DL approaches, the baseline CNN achieved competitive performance, reaching an accuracy of 0.8750 and an F1-score of 0.8727, outperforming both classical descriptors and VGG-16. In contrast, the texture-based CNN exhibited very poor generalization on the independent test set, with accuracy and F1-score values of 0.3333 and 0.1667, respectively. Although this architecture is more sophisticated and, in principle, more expressive, its increased capacity did not translate into improved performance. This behavior can be attributed to the limited size of the dataset and to the fact that the analysis is restricted to regions of interest, which substantially reduces the amount of available training data and increases the risk of overfitting. Under these conditions, the texture-based CNN failed to learn discriminative and generalizable representations.

Similarly, VGG-16, despite leveraging pretrained weights from ImageNet, achieved only moderate performance. This result suggests that transfer learning from natural images may not be fully effective for capturing the subtle texture patterns required for dermoscopic lesion analysis when training data are scarce and domain-specific characteristics dominate.

In contrast, the proposal consistently outperformed all baseline methods. It is important to note that the evolutionary design of the Evo-GMDs was conducted exclusively on the train set using cross-validation, ensuring that no information from the test set was used during optimization. When evaluated on previously unseen data, the Evo-GMDs demonstrated strong generalization capabilities. Notably, the solution corresponding to the median fitness value achieved the best overall performance, with an accuracy of 0.9524 and an F1-score of 0.9231, surpassing both the best and worst evolutionary solutions.

These results highlight an important aspect of evolutionary optimization: obtaining the best fitness value during the optimization phase does not necessarily guarantee the best generalization performance on unseen data. As observed, the median Evo-GMD provided a better balance between precision and recall than the solution with the lowest training fitness, indicating that more stable configurations may generalize more effectively. Furthermore, even the worst evolutionary solution outperformed most comparison methods, achieving an accuracy of 0.9048 and an F1-score of 0.8462, underscoring the robustness of the proposed evolutionary design.

#### Interpretability Analysis and Representative Cases

To further analyze the behavior of the proposed method, this subsection presents representative classification cases that illustrate both successful and failed predictions. Beyond quantitative metrics, these examples provide insight into how the Evo-GMD descriptor captures textural patterns and how similarities in the feature space may influence the final decision. In particular, the analysis focuses on understanding the conditions under which the model is able to correctly discriminate melanoma from benign lesions, as well as the scenarios where overlapping texture characteristics lead to misclassification.

**Figure 6:**
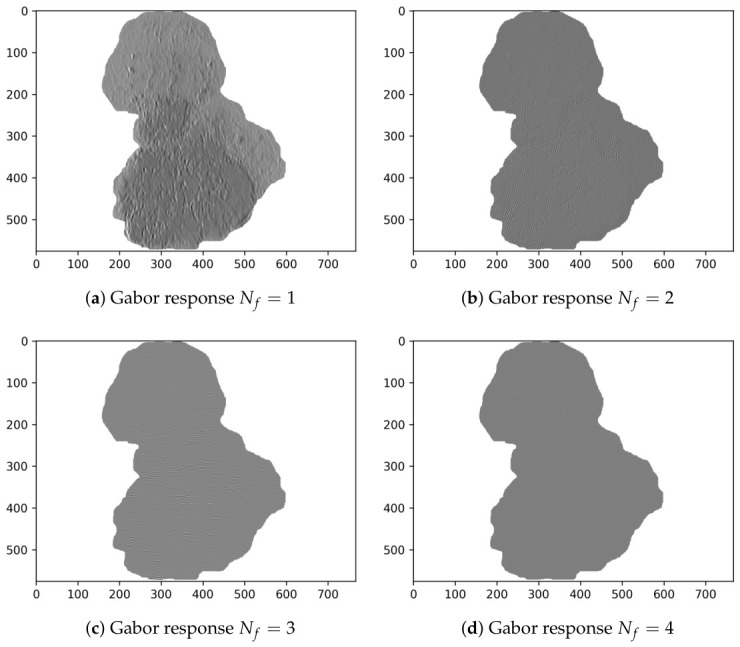
Example of a correctly classified melanoma lesion.

**Figure 7:**
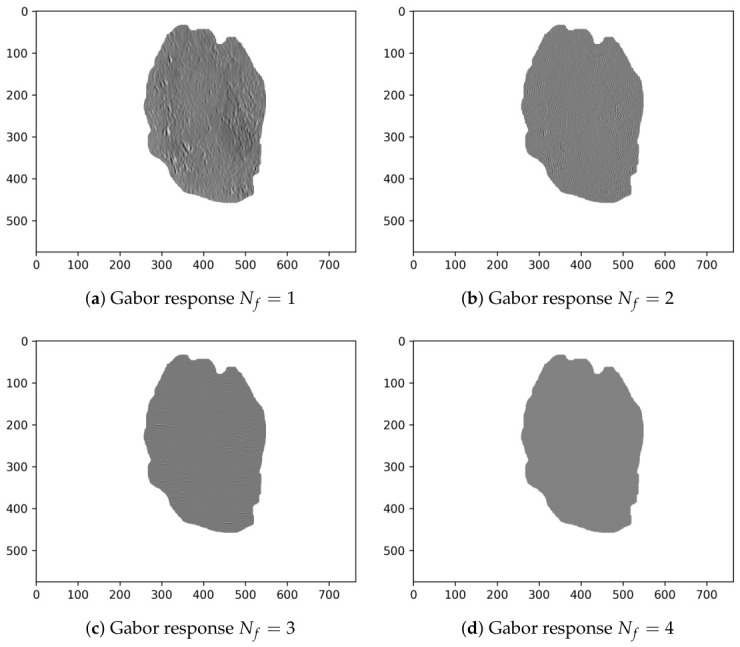
Example of a misclassified melanoma lesion.

Figure 6 and Figure 7 present representative examples illustrating both a correctly classified melanoma case and a misclassified instance.

In cases of correct classification, the proposed Evo-GMD method effectively captures distinctive multi-scale texture patterns that allow melanoma lesions to be differentiated from benign cases. However, misclassification can be explained by the high similarity between the extracted feature vector and those typically associated with common nevi. Specifically, the feature vector of the misclassified melanoma shown in Figure 7 ([327.07, 165.75, −2.36, 17.58, −0.56, 43.81, 0.057, 37.25]) closely resembles that of a common nevus presented in Figure 5 ([350.39, 160.14, −2.45, 22.07, −0.56, 20.60, 0.053, 29.88]). This similarity is particularly evident in descriptors related to mean intensity, asymmetry, and low-frequency texture responses, which play a dominant role in the classification process.

Although differences exist in higher-order texture components, these variations are not sufficiently discriminative within the current feature space to alter the classifier’s decision. In contrast, the correctly classified melanoma shown in Figure 6 exhibits a feature vector ([329.61, 237.92, −2.41, 23.34, −0.61, 23.92, 0.058, 22.48]) that departs more clearly from benign patterns, particularly in terms of intensity distribution and texture magnitude, enabling a more reliable separation.

This overlap in the feature space suggests that certain melanoma lesions may present textural characteristics that are statistically similar to benign patterns, leading to ambiguity at the decision boundary. These observations highlight both the interpretability of the proposed descriptor and its limitations when dealing with lesions that exhibit subtle or atypical texture variations.

### 5.3. Results on the ISIC 2017 Dataset

The performance of the evaluated methods on the ISIC 2017 dataset is summarized in Table 6. In contrast to the results obtained on PH2, all approaches exhibited significant performance degradation, revealing important limitations under more complex and imbalanced conditions.

A consistent pattern was observed across multiple methods, including handcrafted descriptors (LBP and Haralick), the proposed evolutionary approach, and the CNNs. In these cases, the models converged to an accuracy of approximately 80.5%, which corresponds to the proportion of the majority class (non-melanoma). This behavior indicates that the classifiers effectively defaulted to predicting all samples as the majority class, failing to learn discriminative features for melanoma detection.

Interestingly, the texture-based CNN configuration exhibited the opposite behavior, achieving an accuracy of approximately 19.5%. This value corresponds to the proportion of melanoma samples in the dataset, indicating that the model converged to predicting all samples as melanoma. These two outcomes represent degenerate solutions, where the model collapses to a trivial classifier biased toward a single class.

This phenomenon highlights the instability of the learning process under strong class imbalance and increased intra-class variability. Rather than converging to a meaningful decision boundary, the models are driven toward extreme solutions that minimize the loss function without capturing relevant patterns.

The use of transfer learning through VGG-16 provided a partial improvement, achieving an accuracy of 77.33% with a more balanced distribution of precision and recall. However, this performance remains below the majority-class baseline and reflects the limitations imposed by the dataset. It is important to note that VGG-16 benefits from large-scale pretraining, making this comparison inherently favorable to deep learning approaches. In contrast, the proposed method is trained entirely from scratch and does not rely on external data.

Another key observation is the strong dependency of CNN-based models on data augmentation. Without augmentation, these models failed to generalize effectively, either collapsing to the majority class or exhibiting unstable behavior. In contrast, the proposed method maintained consistent behavior without requiring any data augmentation, aligning with the Frugal AI paradigm.

Overall, these results suggest that the primary limitation is not model capacity, but rather the characteristics of the dataset itself, particularly class imbalance and the heterogeneity of the non-melanoma class. Increasing model complexity alone does not guarantee improved performance under these conditions. Instead, the results emphasize the importance of developing methods that are robust to data constraints and capable of operating under realistic clinical scenarios.

## 6. Discussion

Overall, these findings demonstrate that the Evo-GMD effectively captures discriminative texture patterns relevant for lesion classification while maintaining interpretability and robustness. Unlike conventional texture descriptors that primarily model intensity variability through global statistics, the proposed approach explicitly incorporates directional information of textural variations within the lesion, providing a richer representation of dermoscopic patterns. This characteristic is particularly relevant in melanoma analysis, where subtle directional variations in tissue structure and intensity distribution may convey important diagnostic information.

When compared to deep learning (DL) models, whose performance is highly sensitive to data availability, architectural complexity, and training conditions, the proposed approach exhibits superior generalization under limited-data scenarios. The experimental results indicate that increasing model complexity does not necessarily lead to improved performance, as observed with the more sophisticated CNN architectures evaluated in this study. This reinforces the notion that, in small-sample biomedical imaging problems, carefully designed and optimized feature-based representations can outperform data-hungry learning-based models.

To further investigate this behavior, complementary experiments were conducted on the ISIC 2017 dataset, which represents a more challenging and heterogeneous scenario. Table 7 highlights the fundamental differences between the PH2 and ISIC 2017 datasets, providing a clear explanation for the observed performance gap across all evaluated methods. While PH2 offers a controlled acquisition environment with limited variability and well-defined lesion characteristics, ISIC 2017 introduces strong class imbalance, heterogeneous non-melanoma classes (including nevi and seborrheic keratosis), and substantial variability in imaging conditions such as illumination, artifacts, and lesion scale.

Under these conditions, multiple methods—including handcrafted descriptors, evolutionary approaches, and CNNs—exhibited a consistent degradation in performance. In particular, several models converged to degenerate solutions dominated by class distribution, either predicting all samples as the majority class (accuracy ≈80.5%) or the minority class (accuracy ≈19.5%). This behavior highlights the instability of the learning process under strong class imbalance and increased intra-class variability, where models fail to learn meaningful decision boundaries.

Even transfer learning approaches, such as VGG-16, provided only partial improvements despite leveraging knowledge from large-scale pretraining. Furthermore, CNN-based models demonstrated a strong dependency on data augmentation to achieve stable performance, whereas the proposed method maintained consistent behavior without requiring any augmentation. These findings suggest that the primary limitation lies not in model capacity, but in the characteristics of the dataset itself.

Taken together, these results provide experimental evidence that, under imbalanced and heterogeneous conditions, model behavior is largely driven by data distribution rather than architectural complexity. In this context, the proposed method offers a compelling alternative by delivering stable performance without relying on large-scale pretraining, extensive data augmentation, or high computational resources.

Despite the strong performance achieved on PH2, some limitations should be considered when interpreting these results. The experimental evaluation was conducted on a relatively small dataset and focused on predefined regions of interest. However, this setting closely resembles realistic hospital environments, where acquisition costs, ethical considerations, and inter-observer variability often constrain the availability of large, well-annotated dermoscopic datasets. In this context, the ability of the proposed method to achieve robust and generalizable performance under restricted data conditions can be regarded as a practical strength rather than solely a limitation.

Nevertheless, the reduced dataset size and the ROI-based analysis may limit the amount of contextual information available for classification, potentially affecting performance in more heterogeneous clinical scenarios. Future work will therefore focus on evaluating the proposed approach on larger and more diverse datasets, exploring adaptive filter configurations, incorporating color information through efficient fusion strategies, and investigating hybrid approaches that combine the interpretability of handcrafted descriptors with the representational power of learning-based models.

This work demonstrates that effective melanoma classification does not necessarily require increasingly complex models, but rather a careful alignment between model design, data characteristics, and application constraints. This perspective is particularly relevant for real-world clinical deployment, where robustness, interpretability, and computational efficiency are as critical as predictive performance.

## 7. Conclusions and Future Work

This work introduced Evo-GMD, a lightweight and interpretable approach for melanoma classification based on evolutionary optimization of Gabor texture descriptors. The proposed method was designed under the principles of Frugal AI, prioritizing low computational complexity, robustness, and independence from data augmentation and large-scale pretraining.

A wide range of approaches have been proposed in the literature for skin lesion classification, including rule-based methods, CNNs, and hybrid strategies. In feature-based approaches, previous studies have emphasized the relevance of pixel intensity variability for melanoma description. In contrast, the findings of this work demonstrate that not only the magnitude of intensity variations within the ROI is important, but also the directional behavior of these textural patterns.

Experimental results on the PH2 dataset demonstrated that Evo-GMD achieves superior performance (accuracy up to 95%) while maintaining a minimal parameter configuration and full interpretability. Complementary experiments on the ISIC 2017 dataset revealed a substantial performance degradation across all evaluated methods, including DL and transfer learning approaches. In particular, several models converged to degenerate solutions driven by class imbalance, highlighting the limitations imposed by dataset characteristics rather than model capacity.

These findings suggest that increasing architectural complexity alone is insufficient to address the challenges of melanoma classification under realistic conditions, where data variability and imbalance are prevalent. In contrast, the proposed method exhibited stable behavior without relying on data augmentation or external pretraining, reinforcing its suitability for deployment in resource-constrained environments. Likewise, this study provides evidence that effective melanoma classification can be achieved through carefully designed, resource-efficient models, emphasizing the importance of aligning model design with data characteristics and real-world constraints.

Future work will focus on extending the approach to larger and more diverse datasets, incorporating color information through efficient fusion strategies, and exploring hybrid models that combine the interpretability of handcrafted descriptors with the representational capabilities of learning-based methods.

## Figures and Tables

**Table 1:** Parameter configuration for classical texture descriptors.

Descriptor	Parameter Setting
LBP (Uniform)	Radius = 1; Number of points = 8
Haralick	Properties: contrast, dissimilarity, homogeneity, energy, correlation, ASM
	Distances: 1
	Angles: 0, π/4, π/2, 3π/4
	Gray levels: 32
	Symmetric: true; Normalized: true

**Table 2:** Configuration of the DL models evaluated in this study.

Model	Configuration
CNN (baseline)	3 convolutional layers (32-64-128 filters, 3×3, ReLU);
	Global Average Pooling; Dense (128) + Softmax;
	Optimizer: Adam; Epochs = 30; Batch size = 8
CNN (texture-based)	Convolutional blocks (32-64-128) with Batch Normalization;
	Dropout = 0.4; Global Average Pooling;
	Optimizer: Adam ($1\times 10^{-4}$);
	Early stopping and learning rate reduction;
	Epochs = 30; Batch size = 8
VGG-16	Pretrained on ImageNet; frozen convolutional layers;
	Dense (256) + Dropout (0.5);
	Optimizer: Adam ($1\times 10^{-4}$);
	Epochs = 30; Batch size = 8

**Table 3:** Descriptive Statistics of the Fitness Values for 31 Independent Experiments.

Min.	Max.	Mean	Median	Std. Dev.
0.0700	0.1051	0.0918	0.0960	0.0106

**Table 4:** Gabor filter parameters found by DE.

	$N_{f}$	$m_{j}$	$\sigma_{j}$	$\theta_{j}$	$\lambda_{j}$	$\gamma_{j}$	$\psi_{j}$
Median	1	9	4.81270877	3.13540763	7.47685794	0.31561081	2.20899015
	2	8	1.96396977	0.24860970	2.96959010	0.72105363	4.60142864
	3	13	1.64780153	1.61016196	2.83743557	0.66358951	1.73766008
	4	15	3.39179532	0.55921120	1.16027452	0.96851020	0.19780057
Best	1	19	3.12069459	1.51606866	3.89019095	0.65310207	4.58528896
	2	7	3.02631751	0.45433475	5.25153474	0.83115052	2.04598812
	3	8	4.91978266	1.01728566	1.73791384	0.62092611	2.98742533
	4	8	2.76768679	0.29284487	2.12407121	0.50966019	4.89246233
Worst	1	14	2.87441456	0.64689645	7.50065226	0.48449909	0.16186625
	2	9	4.60047050	2.76179466	6.09504738	0.27625496	1.01749004
	3	19	2.91841521	1.58793182	5.60334183	0.17517662	3.61273987
	4	10	1.54570447	1.59859262	2.73917312	0.98337168	1.01393177

**Table 5:** Classification performance on the independent test set for all evaluated methods.

Method	Accuracy	Precision	Recall	F1-Score
Haralick	0.7619	1.0000	0.2857	0.4444
LBP Uniform	0.7619	0.8333	0.3571	0.5000
CNN (baseline)	0.8750	0.8739	0.8750	0.8727
CNN (texture-based)	0.3333	0.1111	0.3333	0.1667
VGG-16	0.8333	0.8125	0.8125	0.8125
Median Evo-GMD	0.9524	1.0000	0.8571	0.9231
Best Evo-GMD	0.9286	1.0000	0.7857	0.8800
Worst Evo-GMD	0.9048	0.9167	0.7857	0.8462

**Table 6:** Performance comparison on the ISIC 2017 dataset.

Method	Accuracy	Precision	Recall	F1-Score	Notes
Evo-GMD (Proposed)	0.8050	0.0000	0.0000	0.0000	Majority collapse
LBP + SVM	0.8050	0.0000	0.0000	0.0000	Majority collapse
Haralick + SVM	0.8050	0.0000	0.0000	0.0000	Majority collapse
CNN (baseline)	0.8050	0.0000	0.0000	0.0000	Majority collapse
CNN (texture-based)	0.1950	0.0380	0.1950	0.0636	Minority collapse
VGG-16 (transfer)	0.7733	0.5649	0.5321	0.5293	Pretrained

**Table 7:** Comparison between PH2 and ISIC 2017 datasets and representative model performance.

Aspect	PH2	ISIC 2017
Total Images	200	2600
Classes	Melanoma/Non-melanoma	Melanoma/Non-melanoma
Class Distribution	∼33% melanoma	∼19% melanoma
Segmentation Masks	Expert-validated	Provided (challenge annotations)
Image Variability	Low (controlled)	High (real-world conditions)
Artifacts (hair, shadows)	Minimal	Frequent
Illumination Variability	Low	High
Lesion Heterogeneity	Low	High (includes keratosis)
Median Evo-GMD (Proposed)	0.9524	0.8050
LBP + SVM	0.7619	0.8050
Haralick + SVM	0.7619	0.8050
CNN (baseline)	0.8750	0.8050
CNN (texture-based)	0.3333	0.1950
VGG-16 (transfer)	0.8333	0.7733

## Data Availability

The original contributions presented in this study are included in the article. Further inquiries can be directed to the corresponding author.

## Abbreviations

The following abbreviations are used in this manuscript:

AI	Artificial Intelligence
CAD	Computer-Aided Diagnosis
CNN	Convolutional Neural Networks
DE	Differential Evolution
DL	Deep Learning
Evo-GMD	Evolutionary Gabor-based Melanoma Descriptor
GFBs	Gabor Filter Banks
HSV	Hue, Saturation and Value
INEGI	National Institute of Statistics and Informatics
ISIC	International Skin Imaging Collaboration
LBP	Local Binary Patterns
MS-GFBs	Multi-Scale Gabor Filter Banks
RGB	Red, Green, and Blue
ROI	Region of Interest
SVM	Support Vector Machine
